# A Thematic Analysis of Students’ Perceptions and Experiences of Bullying in UK Higher Education

**DOI:** 10.5964/ejop.3669

**Published:** 2022-02-25

**Authors:** Emma D. Harrison, Julie A. Hulme, Claire L. Fox

**Affiliations:** 1 Psychology Department, Glyndwr University, Wrexham, United Kingdom; 2 School of Psychology, Keele University, Staffordshire, United Kingdom; 3 School of Education, Manchester Metropolitan University, Manchester, United Kingdom; Edinburgh Napier University, Edinburgh, United Kingdom

**Keywords:** bullying, students, higher education, thematic analysis, focus groups, qualitative

## Abstract

Bullying in higher education (HE) has been relatively under-researched; despite its likely prevalence and impact on student wellbeing there is scant understanding of students’ lived experiences of bullying. We conducted online and physical focus groups with UK HE students (40 undergraduates from 17 UK universities, mean age: 22), exploring their perceptions and experiences of bullying at university. Thematic analysis was used to identify key issues, specifically 1) the importance of a power imbalance and perpetuation of existing systemic inequality in a HE context; 2) bullying in HE is motivated by attainment of social and personal gains; 3) the tactics used to bully in HE resemble those seen in other contexts, but may be more nuanced; 4) bullying can be minimised and justified within HE, leading to its continued prevalence. We conclude that HE bullying shares features in common with school and workplace bullying, and with sexual harassment. However, further research is needed to accurately define and conceptualise bullying in this unique context. HE providers should consider attending to issues of power and inequality within their bullying and harassment policies. They should also ensure there is clear information and guidance to prevent and reduce bullying in universities.

Research into student bullying in Higher Education (HE) has been largely neglected in comparison to bullying in schools, despite growing concerns about student mental health (The Insight Network, 2019). There have been investigations and interventions dedicated to gender-based violence on university campuses (e.g., Fenton & Mott, 2018), but little on bullying behaviour. Students at university are known to experience bullying (Chapell et al., 2006) and bullying across the lifespan is known to be psychologically damaging (Boulton, 2012). However, the lack of research into bullying in HE means that it is not well understood, and no clear definition has been proposed for university students.

School research has defined bullying as a systematic abuse of power where intentionally aggressive behaviour is repeated against a target who cannot defend themselves (Smith, 2004). In the workplace, ACAS (2014, p. 1) suggests that: “Bullying may be characterised as offensive, intimidation, malicious or insulting, behaviour; an abuse or misuse of power through means that undermine, humiliate, denigrate or injure the recipient.” Alternatively, Volk, Dane, and Marini (2014, p. 328) suggest that bullying is “aggressive, goal-directed behaviour that harms another individual within the context of a power imbalance.” Their addition of “goal-directed” attends to motives other than solely the intent to harm that may drive the bullying.

These definitions differ in terms of whether behaviour should be repeated in order to be defined as bullying (ACAS, 2014; Volk et al., 2014), and whether the ability to self-defend is prescribed (ACAS, 2014). These differences may reflect different perceptions of children and adults; perhaps adults are appraised as better able to defend themselves than children. However, this developmental interpretation may be inappropriate, as adults may also be unable to do so. For example, individuals bullied in childhood may be bullied into adulthood (Adams & Laurence, 2011; Brendgen & Poulin, 2018), and learned thinking patterns may persist (Fivush, 2006) leaving an adult victim vulnerable and unable to defend themselves. Likewise, an employee may risk losing their job if they complain about bullying from management. As such, a perceived power imbalance may be more appropriate in definitions for adult-context bullying. Power differences in adulthood are not always visible and may relate to structural hierarchies as well as personal perceptions and social constructs (Prilleltensky, 2008).

Currently, there is a lack of understanding of power dynamics for students within the organisational context of HE. Most university students are in emerging adulthood (EA), possessing characteristics of adolescents and adults (Arnett, 2015), and so it is not clear whether childhood or adult models of bullying are more appropriate. Emerging adult students exist within the organisational structures and social contexts of a university, which offers a different ecological system (Bronfenbrenner, 1979) from the school or workplace. They also tend to be clustered around the 18 to 25 age range, and may experience similar levels of bullying to school children, but more bullying than older adults (e.g., Ševčíková & Šmahel, 2009). This combination of individual and contextual differences between the university student population, children at school, and adults in the workplace may impact the way students and university staff define, classify, and perpetrate bullying. For example, at university there may be more vertical bullying compared to school, where lecturers may abuse their power. It is therefore important to gain a deeper understanding of bullying in HE that accurately represents students’ experiences to inform future research and policy development, and to facilitate interventions to prevent and manage bullying behaviour within HE.

## Aim

The current study aimed to build upon the growing student bullying literature by gathering evidence of students’ understanding of bullying at university. Students are often asked to report their childhood bullying experiences (Espelage, Hong, & Mebane, 2016; Schäfer et al., 2004), but few studies have asked about their current experiences within the university setting. Those that have surveyed students about bullying tend to use small samples and are from the US (e.g., Young-Jones, Fursa, Byrket, & Sly, 2015), Turkey (e.g., Doğruer & Yaratan, 2014), or ask a slightly younger population than university students (Byrne, Dooley, Fitzgerald, & Dolphin, 2016). Some have adopted a qualitative approach, such as Crosslin and Golman (2014), and Brewer, Cave, Massey, Vurdelja, and Freeman (2014), who used focus groups to question US students, but they both limited their interest to cyberbullying only. All studies uncovered conflicting findings in reference to the terms “bullying” and “cyberbullying.” As claimed by Myers and Cowie (2017), in addition to ambiguity around the term “bullying,” student beliefs about bullying in HE need exploring further as many are unaware of the seriousness of it.

The study therefore adopted a qualitative approach to explore students’ perceptions of styles, frequencies, and the intensity of bullying in HE. Examining students’ experiences is critical to understand the nuanced ways in which bullying may differ within HE in comparison to schools, workplaces, and other contexts; qualitative methods allowed students to share their own understandings, without influence from the researcher. An inductive approach was necessary to ensure that the research was genuinely student-led, mindful of student culture (which may differ from that of the researcher; Bronfenbrenner, 1979), and inclusive of experiences of bullying that were not predicted within the literature. A better understanding of bullying within this context could inform more valid measures for use within quantitative studies and inform policies and practice to tackle bullying more effectively within HE.

## Method

### Participants

After gaining ethical approval from the School of Psychology ethics committee, 40 undergraduates from 17 UK universities (16 publicly funded, one independent) participated in focus groups. Thirty-four students comprised four online focus groups from English and Scottish universities, and six students attended a physical focus group at a campus-based pre-92 university. They were recruited via posters published on campus and on social media.

Participants were aged between 18 and 30 years (*M* = 22, *SD* = 2.8), and 28 were female, 10 male, and two undisclosed. Reported ethnicities were as follows: 14 White, 10 Asian, two European, two African, two Caribbean, and two mixed ethnicities. They studied a range of disciplines, all students were single, and all but one was full-time. Individuals in all groups shared student status but were otherwise of mixed demographic composition (as recommended by Hollander, 2004).

### Materials and Procedure

Focus groups are recommended for exploratory research and examining unknown contexts (Frey & Fontana, 1993), and can generate many ideas with only a small number of groups (Morgan, 1997). A semi-structured focus group schedule was created to capture broad views, with open-ended questions such as, “how would you define bullying?.” For the on-campus group, those who emailed their interest were sent an information sheet and they then signed up if they wanted. The group convened in a booked library study room, where a consent and demographic form were completed. After introducing the study and reiterating ethical considerations (including noting the presence of an audio recorder and note taker), we proceeded through the schedule of questions. Participants received a gift voucher for their participation.

For the online focus groups, students expressed interest by email and were provided with the information sheet, consent form, and demographic form (completed before being linked to the focus group platform). On entering the online group (maximum of 10 participants), participants answered the same interview schedule questions as used with the face-to-face group. They were able to respond to others’ replies and to the moderator, and to add extra comments later. Once all the questions had been answered, participants were reminded to complete their responses prior to the imminent closure of the group. Following participation, participants were debriefed and invited to claim an electronic gift voucher. This process was repeated for four online groups.

### Analysis

Thematic Analysis was chosen to analyse the data as it is flexible, accessible, and not aligned to one theoretical framework (Braun & Clarke, 2013). A middle-ground approach was adopted between essentialist theory and a social constructivist analysis; analysis was primarily data-led, but influenced by our knowledge of existing literature, theories, and definitions from other contexts, as well as our experience of HE and personal understandings of bullying. Recognising our subjectivity and our generational perspectives (Bronfenbrenner, 1979) was important within the analysis, and valid interpretations were managed throughout by checking across members of the research team.

The first author familiarised herself with the data through repeated listening to the audio recording and multiple readings of the online group data. She then verbatim transcribed the audio data and copied the online data into document format, annotating the data with reflections in the margins. Codes (units of meaning) were then generated line-by-line for all data. Following coding, codes were then grouped together under broader umbrella themes. Similarities were noted between verbal, physical, psychological, and cyber bullying from the school literature. The codes were checked for credibility by the second and third authors, who also identified some connections between the data and the relationship abuse literature.

## Results

The themes and subthemes can be seen in Table 1. The first theme identified was that bullying involves a power imbalance between social groups. Next, the data were interpreted to suggest that perpetrators bully for either social or personal gain. Thirdly, students reported some common bullying tactics. The final theme suggested that bullying is maintained by inaction and the justification of bystanders. Note that quotes are verbatim but corrected for spelling to aid clarity.

**Table 1:** Main Themes and Subthemes Identified From the Focus Groups

Main Theme	Subtheme
Power imbalance	Social groups
	Status and reputation in the social hierarchy
Objective of bullying	Intentional and goal-directed for social gain
	Intentional and goal-directed for personal gain
Methods of bullying and tactics used	Sexual harassment
	Active exclusion and isolating
	Online/cyber
	Controlling and mind games
	Verbal and jokes
Justification and minimisation for involvement in bullying	Bystander intervention

### Power Imbalance

#### Social Groups

Students reported that bullying happens between groups or where groups attack an individual: “social groups will pick on an individual who they think is 'less intelligent' than the rest and make that person the butt of most of their jokes” (Online Focus Group 3 participant 4; OFG3.4). Individuals within certain demographic groups may also bully, sometimes based on group-level differences like ethnicity and class: “I think bullying comes mainly from majority groups towards minority groups in higher education. For example, from those from private education/more privileged backgrounds towards those from less well educated/less privileged backgrounds” (OFG1.9). Likewise: “Yeah, have seen students of different races in an argument in the library because of a derogatory term being used from one party to the other” (OFG1.7) and “…but same goes with verbal, which can occur because of someone's ethnicity or race, even sexual orientation” (OFG3.6). Those who are in a majority or privileged group seem to have more power than those in minority or less privileged groups, which could be advantageous in many ways: “… and confident people tend to come from good socioeconomic backgrounds and have support” (OFG4.3).

The power may be so ingrained that those who are bullying do not realise they are doing it: “I would say verbal bullying mainly includes racism and discrimination in LGBT group. Sometimes people probably won’t even notice what they do to others is actually bullying” (OFG3.8). In general, there was agreement that minoritized groups were more likely to be bullied: “…but perhaps those bullied are often minorities or have been unfairly and inappropriately portrayed in a negative light from other sources” (OFG2.5). It may be that those who bully do not recognise their privileged positions or understand the effects of their actions.

#### Individual Status and Reputation in the Social Hierarchy

Individual power may also come from position in a social hierarchy. Students agreed that teachers and lecturers can bully students, abusing their higher position in the classroom and authority over the students. One person said: “Teachers could also be included in the bullying, when they take part in humiliating a person or picking on them constantly in class or talking aloud when making comments about a student's work, conduct, activities or indeed appearance” (OFG3.3). This could bolster a teacher’s existing power whilst lowering the target’s power and reputation publicly. Some students claimed an equal relationship with staff at university, although others disagreed, feeling that lecturers had more power than students:

So I know a friend, so she feels like she’s been bullied by a lecturer [moderator: ok], so I feel like that’s different than at school when you wouldn’t really consider it to be bullying by a teacher [moderator: no] cause you’re kinda more equal here (Physical Focus Group, participant 5; PFG.5).

This implies that students have expectations of lecturer behaviour based on the lecturer’s authoritative role. As well as job role, there are other personal characteristics that perpetrators shared that granted relative power and reputation. For example, attractiveness was mentioned: “They are people who appear outwardly confident and they're usually stylish, attractive and have an entourage” (OFG1.4), and confidence: “Often more confident individuals are the perpetrators, especially if they have settled in quite quickly and easily” (OFG4.2). Having a sociable personality increased the likelihood of popularity, which in turn linked with confidence: “A person who is a social butterfly will have less chance of getting bullied because they appear more confident” (OFG2.3) and: “It’s always the one doing the bullying who is ‘popular’ and they rally support from unconfident people who they allow into their group” (OFG1.4). These characteristics were thought to be associated with status and perpetration.

Perpetrators may attempt to damage social status and reputation, which can be important factors in students’ lives: “The bully manipulates the victim’s social status by spreading rumors or ostracizing the victim from his or her peers” (OFG2.1). By lowering another’s social status, their own may increase: “…they want to look the ‘big man’ and show off” (OFG3.3). Acquiring more power may be a motive for bullying as the power will increase their social status: “When they have power over someone else it gives them a superficial sense of authority” (OFG1.4).

### Objective of Bullying

#### Intentional and Goal-Directed for Social Gain

Participant discussion suggested that bullying is intentional and goal-directed, with some occasional conversation about being hurt unintentionally through ignorance. Bullying is perceived as social and thus socially motivated: “People like the validation of others and joking around, teasing and singling somebody out is an easy way of bonding with others at the expense of the one they are making fun of” (OFG3.5). Students may join in with bullying to bond and fit in or avoid becoming the next target: “Wanting to fit in with other students—if the bully knows others feel the same way about the target, it may be a way to bond” (OFG4.2), and: “Though if one person starts something, other people may join in” (OFG1.6).

#### Intentional and Goal-Directed for Personal Gain

Bullying may also be linked to personal gain, especially in one-to-one situations: “It is a complex issue of which perpetrators bully people for their own gain for different reasons. It could be a number of things—their own weaknesses, attention, jealousy, dislike to the victim” (OFG2.9). If a student feels weak, bullying another may bolster their own self-esteem and feelings of power and self-efficacy. Perpetrators may desire agency and control over others: “Yes, once you respond it just fuels the bullies’ desire to gain total control over you” (OFG1.7), and: “You have people who get a sense of power from limiting others from joining in” (OFG1.4). Instead of, or alongside, a social goal, the purpose may be to control the environment and the people within it to make themselves feel better. Perpetrators may attempt to assuage their insecurity by attempting to become superior: “It's intrinsic for humans to want to be superior (especially those people who are actually secretly insecure inside)” (OFG1.7).

### Methods and Tactics of Bullying

The third theme shows how most students had ideas about how bullying is perpetrated at university (even if they claimed not to have witnessed it), which suggests a shared social representation based on beliefs.

#### Sexual Harassment

In three of the focus groups sexual harassment was commonly discussed as being a problem, with many experiencing it themselves or knowing others who had: “Aggression directed at female students / sexual harassment (i.e., groping, making unwanted sexual remarks)” (OFG1.3). Many thought this was harmful: “There is sexual harassment, which appears a lot more subtle from outside but I think if you’re a young woman who gets that sort of overt interest it can be quite uncomfortable” (OFG1.4). Most thought it regularly happened to women, but one student noted men can experience it too:

It does happen the other way round, but I feel that it isn't as prevalent. This isn't a reason to ignore it. I'd say 90% of my female friends have experience of men in clubs groping them, and maybe 40–50% of my male friends. When it gets more sinister like following you home, forcing themselves on you, or pulling your skirt up/top down, I find that men haven't had to deal with this, but a number of women have (OFG4.5).

This quote indicates a widespread gendered issue. Sexual harassment may not typically be perceived as bullying, but students did categorise it as such in this study: “Besides that, I believe that frequent catcalling, sexual abuse, it all counts as bullying” (OFG1.2).

#### Active Exclusion and Isolating

The group tactic of excluding or isolating individuals was a commonly discussed method within universities. This could be in the form of an online chat box or in person: “…so the group would make subtle remarks about them or talk about them in a group chat” (OFG3.1); “Active exclusion which takes a negative form. Often takes place in social groups—excluding one person who you live with from social events, one person in your lectures you actively move away from” (OFG4.2). Not only can this be hurtful, it may also affect work: “Exclusion from group projects and ignoring peers and people in their groups, leading to unfair exclusion from university work which may lead to lowered grades” (OFG4.3).

Students also discussed how conscious people were of excluding others and whether an active decision to exclude was needed to classify it as bullying. As one student said: **“**We don't have to be friends with or include everyone” (OFG4.5), suggesting that they may perceive excluding a stranger as acceptable. However, perhaps persistent exclusion or behaviour that follows exclusion, such as socially encouraged bad behaviour, could re-classify exclusion as bullying:

Being excluded from groups and purposely ignored could also be thought of as bullying however it is a person's right not to want to speak to someone, but when you then turn others against a person with no due cause, this is bullying (OFG3.3).

This student suggested that not all exclusion can be classed as bullying but if the excluder then turns other people against the excluded, it is a bullying act. Another student gave anecdotal evidence to support this: “Agreed, my flatmates in first year did this. Excluded a girl in our flat for no reason along with being nasty and cruel, resulting in her being very upset” (OFG4.3).

Another person claimed that exclusion was not even an issue because they had not witnessed it: “I believe that there might be more pressing matters than exclusion/bullying (since I haven’t witnessed it yet)” (OFG4.4), whereas another participant suggested that bullying can lead to exclusion: “I think exclusion can happen as a result of bullying, I would not say exclusion is part of bullying” (PFG.1). Consequently, the tactic of targeted exclusion may be an initial step in bullying an individual.

#### Online/Cyber

The discussion surrounding cyberbullying addressed different perspectives, suggesting it was a strong but controversial theme. Students provided multiple examples of cyberbullying: “…it can either be passed around the group chat for everybody to laugh at, posted publicly on Facebook or even used as blackmail” (OFG3.4), which suggested the internet could be a channel for various types of abuse. Students were aware that harm can be caused by bullying online: “cyberbullying can involve many different types of bullying such as sexual harassment, racism, sexism and homophobia. Saying something nasty, cruel or offensive online is no different than saying it in person in terms of the victim's suffering” (OFG4.3). Cyberbullying allows for simple perpetration: “Everyone is connected online pretty much 24/7 these days so cyberbullying can take place anywhere” (OFG3.5), and is easy to hide: “I believe that Cyber Bullying deserves special attention, since it is much easier to commit due to increased anonymity” (OFG4.4). One person suggested that online bullying could be an extension of traditional forms of bullying: “For example, someone may be excluded from social events at their halls and yet may be involved in cyberbullying at the same time” (OFG4.6). The visual content of social media can be particularly damaging: “Online bullying through nasty messages and sharing of private information/photos would be a devastating method becoming more common through the rise of snapchat and other photo-centric social media apps” (OFG4.6).

Alternatively, some claim cyberbullying does not happen at university: “I’ve not seen much cyber bullying in the university context” (OFG4.5), and: “I have never experienced cyberbullying and have not heard of cases of cyberbullying in university within my group of friends and acquaintances. For many students, I think cyberbullying is not that big of an issue” (OFG3.5). Other students approached the subject from a neutral position suggesting they may not have seen it because it is rare or covert, not because it does not happen: “I don’t think cyberbullying is a big problem at university. If it is, students are very secretive about it, and, in my opinion, it cannot be seen online, so it would have to occur through messages” (OFG1.2). Another student said:

In higher education bullying can be more complex and is rather in the verbal or written form with cyber bullying being more prominent. Even though I’m saying that there are forms of bullying in higher education, I have rarely witnessed it at my university, but then again the whole point of cyber bullying is that it’s silent and invisible (OFG3.4).

The data suggest that the internet may be a frequently used and damaging tool for bullying and harassment in HE, in part because of its invisibility.

#### Controlling and Mind Games

Those who bully were reported to use intentional tactics to control a person or their environment. Control could be exercised through actions: “It's usually verbal abuse, but also actions, such as listening to loud music on purpose when the bullied person has an exam in the morning or throwing away their food” (OFG1.2). The perpetrator controls the environment by creating disturbance or stealing possessions. One student suggested it is like playing mind games: “I think it is a psychological abuse in trying to play mind games with you [moderator: right] rather than getting on with what you’re here to do” (PFG.1). Other students said similar, suggesting the control could come in the form of pressurising: “I think bullying could be mentally manipulative, using a dominating nature to force someone to do something in their favour” (OFG3.7).

Owning or having control of a situation was identified as a motive for bullying (see previous theme) as well as comprising a bullying tactic: “…people just like to have power/control over others and do not care about others’ feelings” (OFG2.7). One student commented:

A lot of people can lash out to others because they are in control of their own actions, they can control what they say to people and they can see what reaction they get from it, so, a lot of people will deliberately do things because they feel in control of it (PFG.6).

Another participant in the physical focus group agreed that control was the motive for bullying as well as being a tactic to control the environment, providing reasoning for how bullying is difficult to address:

…it’s difficult to resolve, because there’s things that people put across to try and counter bullying…the irony is, the people that are doing it are often deaf to seeing reason in that sense and seeing that they are causing harm [moderator: hm hm] especially if they mean it, because as we’ve mentioned before, it’s a sign of control, of something they can do that empowers them (PFG.1).

It appeared that students were clear that a bullying motive was to gain control, and that controlling a person, or their environment, was a method of bullying. This shows that control is an integral and entrenched aspect of bullying.

#### Verbal and Jokes

There were many examples of verbal bullying in the form of name calling and making jokes at others’ expense: “Some people try to pass it off as a joke to feel clever” (OFG1.6). One student indicated the confusing nature of knowing when to laugh along with harmful jokes:

Like a racist joke sometimes can be a type of bullying as I found it's not funny at all, but this actually happened a lot in conversations with “friends”, you never know if they are actually being funny or they are just using a funny way to hide their bullying. I personally have some experiences with these racist jokes, the boundary is very vague (OFG3.8).

The boundary between joking (i.e., banter) and bullying is vague, with other students suggesting that jokes are harmless but it depends on interpretation: “Most bullying I’ve seen if you can call it that has been light teasing and generally harmless but some individuals might find it more harmful than others” (OFG3.1). In one group there was some disagreement about teasing with one person saying: “I think its harm depends on its severity. I would not call teasing bullying” (OFG4.3), and another suggesting that: “…it depends on the context. Repeatedly teasing someone for, for example, their appearance can be devastating. A joke among friends is something entirely different” (OFG4.4). It seems that who is doing the teasing is an important factor: “This may be seen as light teasing, but it does obviously have an effect on a person, especially if they thought it was their friend” (OFG3.4). Having a friend who harmfully teases may suggest that the boundaries between friends are unclear: “It can happen within a group of friends when some people think they are just joking around but then one person feels ostracized all the time but does not really speak up” (OFG3.5). If the perpetrator is a stranger, it may be easier to infer they intend harm than if the perpetrator is a friend. Similarly, a lecturer making a joke at one student’s expense may be seen as bullying if the staff member is not mindful of the student’s boundaries: “Whoever is leading the taught session joining in with a joke being shared at the expense of another would be bullying as well” (OFG1.8).

### Justification and Minimisations for Involvement in Bullying

The final theme related to the minimisation of bullying and justifications for not getting involved. It seems that students’ beliefs about bullying, identifying it, and not knowing whose responsibility it is to intervene allow it to continue. Some students said they would get involved but the majority preferred to avoid it. Failure to intervene shows implicit acceptance of bad behaviour by allowing it to continue. Some students knew of bullying incidents or had heard about them and so could have chosen to act. Their option in that situation would be to help the target, tell someone else, or do nothing. Some justified not getting involved due to fear: “I probably wouldn't interfere, especially if it's a heated argument. You'd never know if the parties could get violent. Don't want to get involved with that” (OFG1.7). Fear could be associated with the people involved in the conflict:

If the person that was targeting somebody was a big bulky male that seemed to be very aggressive, most people would be deterred from intervening, whereas if it was, a, if it was a smaller female that’s kind of bitching about something perhaps they’d be more likely to intervene (PFG.1).

Fear can be an understandable reason for lack of involvement, especially if the onlooker has low self-esteem or feels unable to make change without exacerbating the situation or becoming a target themselves. However, not everyone felt the need to justify bystander behaviour because they thought it was not their responsibility to get involved: “The person being bullied should learn to stand up for themselves” (OGF1.7). Another student echoed this: “I don’t think the issue needs intervention because most of the time the victim of bullying has the maturity to walk away, or confront the bully at this stage in life” (OFG3.4).

Additionally, students first must decide whether the situation warrants intervention before they intervene, if they want to. If the aggression seems ambiguous (e.g., jokes) or covert, it is difficult to know the right course of action: “If I don't know someone or you just see people messaging each other about another person, I don't really know what I would do or how you should react” (OFG4.5). One student reiterates the difficult line between banter and bullying: “Sometimes you see things but I don’t know if it’s just classed as like banter between friends” (PFG.5). The onlooker must first feel confident to decide what is or is not bullying, they then need information about what to do and must believe they have the power to change the situation. With this high cognitive effort, it may feel easier to downgrade the importance of a situation, thus absolving themselves of responsibility: “Name calling definitely happens, but nothing major ever happens where someone can intervene” (OFG3.4). This assumptive attitude can also be seen in one student who shows a disinterest in others’ problems: “Most people probably ignore it and assume that as adults everyone can handle their own problems” (OFG3.2).

#### Bystander Intervention

A subtheme of bystander intervention was identified after drawing together the issues of onlookers; they may have the power to step in if they avoided minimising or justifying their reasons for not doing so. Some students said they felt empowered and able to intervene: “Before I probably wouldn't get involved, but today I'm much more mature and confident in myself and would try to stop it” (OFG3.6). In contrast, another student felt that intervening was a moral issue: “It's dependent on how comfortable the person feels about their own role in the group before they intervene. I don't really care about that type of thing, so I tend to just act on what I think is right” (OFG1.4). Some students claimed they proactively help others: “I usually go and sit with the excluded person and my own friends join me” (OFG1.4), and “If I see people who I know being bullied verbally I typically say something” (OFG4.5). Whether a student intervenes may involve several factors including whether the perpetrator or victim is known: “If the bully is someone I know, I would immediately intervene and make them stop” (OFG1.6). However, it is impossible to tell whether these students are offering socially desirable responses, or whether they genuinely intervene when witnessing bullying.

## Discussion

This was one of the first studies to investigate students’ perceptions and experiences of bullying in UK HE, and identified some important themes. The strong theme of power resonated with the existing bullying literature; power was described as existing through social group membership or being gained through bullying. Increased power was associated with a higher reputation in the social hierarchy. This supports evolutionary theories; those who bully can be intelligent, resourceful, and without emotional deficiencies (Volk, Camilleri, Dane, & Marini, 2012). People may bully to gain resources which is a continued incentive for the bullying. This maps onto the second theme—reasons for bullying, which included social or personal gain. This is consistent with Volk et al.’s (2014) definition of bullying as “goal-directed.” Thirdly, tactics and methods used were evident. Some matched school-bullying types, but others were more mature, showing connections to abusive control in romantic relationships. Lastly, students attempted to justify why they would rarely intervene in bullying situations involving strangers and minimised the situation to seem less serious.

Consequently, having or gaining power grants advantages, which are maximised through the tactics employed. Those who witness bullying incidents must decide whether to intervene. Minimising bullying or justifying non-involvement can inadvertently reinforce bullying and normalise the behaviour. Participants described how this can lead to perceptions that victims do not need helping, or they ought to help themselves, which can prevent bullying from being addressed.

### Power

Students described visible power imbalances within their social environment and reported that some individuals actively pursued goals to gain increased power. These data are consistent with Smith’s (2004) and Volk et al.’s (2014) conceptualisation of bullying as aggressive goal-directed behaviour harming another and encompassing a power imbalance. Our study suggests that power is derived from group membership and possession of externally positively evaluated characteristics (which intertwine, e.g., being a member of the white male group), and roles. These power imbalances within the social hierarchy in HE could fuel bullying.

According to Pratto, Sidinius, and Levin’s (2006) Social Dominance Theory, power is inherent within certain societal groups, can be granted by maturity (i.e., lecturers and students), male gender, and arbitrary systems such as ethnicity and social class. Visible group characteristics such as ethnicity and gender (Link & Phelan, 2001) create power differentials and thus allow structural and individual discrimination within universities (Prilleltensky, 2008). Some of the students from the focus groups had direct experience of racist jokes and sexual harassment, highlighting the need to be mindful of the implications of individual and group-based power differences in understanding and researching HE bullying.

Thornberg (2011) suggested that groups label other groups as deviant, leading to stigmatisation of lower status social groups and perceptions of deviance. Our data, emphasising social group involvement in bullying, suggest that as well as structural power differences such as ethnicity, gender, and class, other privileged aspects (attractiveness and popularity etc.) are important predictors of bullying behaviours at university too. The findings support results from previous research; for example, Lund (2017) describes the social exclusion of a class member described as “weird,” indicating unpopularity. Students in our study mentioned attractiveness and being a “social butterfly” as advantageous characteristics that may be associated with power, possibly due to a halo effect (Nisbett & Wilson, 1977). This is where a blanket positive evaluation is given based on one or more appealing traits. For example, Talamas, Mavor, and Perrett (2016) found that faces rated as more attractive were also rated as more intelligent. Such external traits may produce biased perceptions of power. Members of socially dominant groups enjoy positive social value (Pratto et al., 2006) and are awarded more social resources.

Therefore, individuals with confidence, extroversion, and attractiveness may be aware of the power they hold (Prilleltensky, 2008) or bully unintentionally because of the normalisation of their privilege. Alternatively, being black, female, or low socioeconomic status (SES) could disadvantage because of global negative evaluations fuelled by damaging stereotypes (Link & Phelan, 2001). Pre-existing power seems to facilitate bullying. This in turn exacerbates the power differences, producing a group-based hierarchy on multiple-levels, encompassing discrimination from institutions, individuals, and intergroup processes (Pratto et al., 2006). Our findings suggest that perpetrators may be unaware of their privilege, and thus fail to recognise their behaviour as bullying.

In terms of different roles, not all members of privileged groups are active perpetrators; our participants mentioned “entourages” and “supporters.” However, in assimilating into a group, indirectly involved individuals may adopt the group norm that bullying is acceptable (Srivastava, Guglielmo, & Beer, 2010). These individuals may reinforce their identity at an intermediate level as a member of a social in-group defined against an out-group through supporting bullying behaviour (Hornsey, 2008). As well as group-based roles, role-based authority also confers power, and this is true for lecturers in HE. This could be due to the age-based aspect of Pratto et al.’s (2006) Social Dominance Theory, or due to their apparent “expert” status; they award grades, and exercise power over students’ eventual outcomes (Alsobaie, 2015; Hulme & Winstone, 2017). Our participants reported instances of lecturers bullying students and framed this as an abuse of power.

Although membership of a minoritized group brings disadvantage and identifying with a socially privileged group brings individual and group power (Turner, Oakes, Haslam, & McGarty, 1994), groups do not always behave homogeneously. But like other bullying research, this study confirms that a power imbalance is an important factor in the HE context, and that higher status groups may maintain their position of power in the hierarchy through bullying lower status groups.

### Objective for Bullying

Students claimed that bullying in HE is goal-directed for personal or social gain, supporting the definition by Volk et al. (2014). Social goals include group membership and popularity and can be personal or for the benefit of the group. Perpetrators may validate each other and bond by targeting the same individuals, potentially increasing feelings of belongingness, perhaps especially for the entourage who may feel insecure.

Our findings are consistent with Salmivalli’s (2010) claims that motivation by social goals would be apparent in situations where peer status is important. Reputation is important to university students and social motivations are apparent in our data. One person said that those who bully manipulate the social status of others. Perpetrators may attack those with less existing power to maintain their reputation or they may join a powerful group to claim power or reflect their need to belong.

Bullying may also be motivated by personal goals, possibly to increase the perpetrator’s self-esteem by lowering that of the victim. One student mentioned that this type of “mental manipulation” can occur even between friends. This idea of increasing personal power through belittling others resonates with Volk et al.’s (2012) conceptualisation of bullying for advantages or resources.

Control of others was also noted to be a personal goal of bullying in HE, and shares features in common with abusive romantic relationships. Having control over a partner can be achieved in many ways, for example, financially exploiting, verbally harassing, or sexually abusing (Domestic Abuse Intervention Project, 2011). Overlaps between bullying and sexual harassment and violence have been previously noted, with some shared perpetrator characteristics (Basile, Espelage, Rivers, McMahon, & Simon, 2009). Given our findings that bullying in HE is associated with control, it may be interesting to further explore whether similar objectives may exist within HE bullying and relationship abuse.

### Tactics and Methods Used to Bully

#### Sexual Harassment

Sexual harassment, mainly from males towards females, was a commonly reported tactic in this study, perhaps unsurprisingly, as literature reveals that sexual bullying happens even amongst school children (Gruber & Fineran, 2016). There has been widespread media coverage and plans for tackling sexual harassment at UK universities; recent articles allege that around half of students face unwanted sexual behaviour (Batty, 2019; Batty & Cherubini, 2018).

In relations to Basile et al.’s (2009) findings, our findings highlight an overlap between bullying and sexual harassment, supporting the idea that perhaps “neither form of peer violence is simply unilateral” (Hertzog, Harpel, & Rowley, 2015, p. 22). Childhood bullying could be a precursor to later sexual or relationship abuse indicating a continuum of aggressive behaviour using power, which is included in bullying definitions and was a theme within this research. Thus, those who bully peers in school may transfer aggression throughout education and to other contexts, suggesting a trajectory of perpetration using power (Monckton-Smith, 2020).

#### Active Exclusion and Isolation

Power imbalances are also pertinent to bullying through excluding someone from group activities or group work. Research shows that indirect or relational bullying increases with age. Archer and Coyne (2005) suggested that covert relational bullying is an alternative to direct aggression as the legal and social consequences of using direct, physical aggression are too high for adults.

There was some ambiguity as to whether unintentional exclusion was bullying. Similar themes exist within the childhood literature. For example, Killen and Rutland (2011) stated that exclusion is not always a moral transgression; someone excluded from a sports team because they are not sporty may not experience bullying. However, the victim may experience harm regardless of the excluder’s intentions (e.g., see experimental research showing the negative effects of ostracism; Hartgerink, van Beest, Wicherts, & Williams, 2015). Exclusion, discrimination (Sinkkonen, Puhakka, & Meriläinen, 2014), cliques, and ostracism (Brock, Oikonomidoy, Wulfing, Pennington, & Obenchain, 2014) have been reported elsewhere in the HE literature. Our data support this literature, with students reporting exclusion in lectures (people move away from certain students), when conducting group work, and in group chats. University provides many opportunities for exclusion and this is especially likely for minoritized and discriminated groups, emphasising a need for inclusion campaigns at university.

#### Cyber and Online Bullying

Our participants reported witnessing or experiencing cyberbullying at university, although there was some debate about what is acceptable behaviour and what is bullying. This is consistent with Walker, Sockman, and Koehn (2011) who found that a third of their sample had experienced undesirable online behaviours but that participants did not class it as cyberbullying. Our participants said it would be easier to perpetrate because of the anonymity and disassociated nature. In common with social exclusion tactics, some ambiguity around cyberbullying may arise from uncertainty regarding intent. Students from Crosslin and Golman’s (2014) sample said that the sender had to intend harm to be cyberbullying, but this may be unclear in online interactions.

Those who initially claimed cyberbullying did not happen at university subsequently suggested that its covert nature may make it invisible. This indicates that cyberbullying does happen but may not often be witnessed by outsiders. Cyberbullying has been widely reported; Wolke, Lee, and Guy (2017) suggest that most victims of cyberbullying experienced traditional bullying as well, and that cyberbullying extends the bully’s power and control beyond the school yard or university campus to intrude into their free time and personal space. Therefore, even though cyberbullying is covert, it may be that those who are victimised traditionally at university are also the cyber victims, and so the students who indicated it does not exist may never have witnessed it.

#### Controlling and Playing Mind Games

Control was a recurring word used by participants to discuss tactics as well as motivations for bullying. They described the tactic of control as an attempt at commanding others’ agency or esteem. Coercive control as a construct is a central feature of many conceptualisations of domestic violence, although there are variations in how it is defined and measured. To coerce is to control a person’s feelings and/or behaviour. For example, controlling a person or their environment is a key feature of the Duluth model of power and control (Domestic Abuse Intervention Project, 2011), developed to educate about the features of relationship abuse. These include coercion, threats, intimidation, pressures, emotional and economic abuse, isolation, minimising, and denying. This model has been adapted to conceptualise workplace abuse and bullying and matches examples from the current HE study. For example, participants discussed cruel looks, disposal of food, name calling, humiliation, mind games, and isolation. Alongside this, a review by Public Health England (Fenton, Mott, McCarten, & Rumney, 2016) on preventing sexual and domestic violence in UK universities featured much evidence of sexual coercion, with predominantly female targets.

Within the Duluth model, minimising and denying reflect victim-blaming tactics by perpetrators linked with control. Our participants who witnessed bullying also sometimes justified it or blamed the victim, for example, suggesting that the victim should have the “maturity” to walk away or confront the bully. This shifts the responsibility from the perpetrator, who created the situation, onto the victim, who was unwillingly subjected to abuse. Victim-blaming may have become a common discourse, which has normalised the behaviour and lessened the collective perception of the seriousness of bullying, explaining why bystanders may also adopt this approach.

Both versions of the power and control model (Domestic Abuse Intervention Project, 2011; Scott, 2018) talk of privilege in the forms of male privilege and employer privilege, respectively. Students in this study believed that privilege exists in HE in the forms of ethnicity, sex, class, and economic resources. Privilege could also be afforded to lecturers who are in a position of authority.

#### Verbal and Jokes

Verbal bullying is one of the four types of childhood bullying (Björkqvist, 1994; Olweus, 1993; Wang, Iannotti, & Nansel, 2009) and has been reported in previous university studies (Doğruer & Yaratan, 2014). Similarly, unwelcome name-calling and joking were witnessed by our participants, who sometimes found it difficult to define the boundary between banter and bullying. Perpetrators may defend verbal bullying by re-categorising it in a more socially acceptable way as “only joking” or “teasing.” Such teasing was sometimes described as harmless, and sometimes as hurtful, even from friends, especially when hurt cannot be communicated to the joke-teller. Perpetrators may deliberately obscure their intentions to maintain their social reputation. The pretext of “only joking” minimises the act, leading the target to question their own reactions to being a target of banter or jokes. Verbally ambiguous harassment may deceive onlookers into believing the perpetrator means no harm, thus allowing them to continue the behaviour and own the situational power. This type of minimisation is outlined in the next section. Further, if the victim verbalises their concerns, they may experience victim-blaming (as described above), leading to self-blame and shame.

### Justification and Minimisation of Involvement in Bullying

Our data suggest that students may minimise and justify bullying, often through relabelling it as banter, which allows it to persist without consequence or intervention. Some students who were aware of bullying were reluctant to get involved because they were afraid of being similarly victimised. Others were adamant that bullying was not an issue, and if it were, it was not their responsibility to be involved, representing either a moral disengagement or a belief that students (as adults) can tackle things themselves. Miller et al. (2019) found evidence for members of staff in HE institutions morally disengaging from bullying and violence through using justifications, euphemistic language, and diffusion of responsibly. Similarly, Thornberg, Daremark, Gottfridsson, and Gini (2020) conducted research with Swedish school children; using hypothetical scenarios, they found that moral disengagement was higher if the victim was perceived as mean, and moral justification for bullying was higher if the victim was perceived as mean and was surrounded by a laughing group. It may be that comparable mechanisms apply to students in HE.

Remaining a silent bystander lends implicit approval to the situation (Randall, 1997) contributing to the wider societal problem. The victims seem to have been classed as “other” in an out-group who ought to sort their own problems, eliminating personal responsibility from onlookers. Stigma theory (Goffman, 1963) suggests that once labelled as deviant or different by the beholder, the stigmatised person or group transcends taken for granted norms. If a bullied person becomes stigmatised, the norm of helping those in need is irrelevant.

Some students wanted to help but there were barriers preventing them. They were unsure whether it was really bullying, feeling they had insufficient information to make a rational decision, and uncertain about what to do. The decision-making process maps onto Latané and Darley’s (1968) seminal bystander model, which has since been built upon by school bullying researchers who have focused on the role of the group in bullying incidents (Salmivalli, Lagerspetz, Björkqvist, Österman, & Kaukiainen, 1996). The approach has been influential in recognising the role of social context in promoting and maintaining bullying. In a longitudinal study, van der Ploeg, Kretschmer, Salmivalli, and Veenstra (2017) found that a higher level of affective empathy and self-efficacy predicted defending behaviour. Children have been found to be quite aware of the benefits and costs of intervening in bullying situations (Spadafora, Marini, & Volk, 2020); costs included getting into trouble, loss of friends, loss of popularity, and becoming a target themselves. Our findings indicate that similar costs may be perceived by university students. Research on bullying in HE would benefit from school bullying insights to identify the factors that influence why students do and do not intervene.

Additionally, after interviewing 51 university students about bystander interventions, Holtzman (2020) concluded that students who had received bystander intervention training deemed bystanders responsible for intervening. However, on being presented with vignettes where the bystanders did not intervene, students provided excuses for why this might have been the case (e.g., gender, lack of knowledge, and friendships with perpetrators). Most workplace and university anti-bullying policies encourage witnesses to intervene, and it seems students are aware of the importance of intervening, but intervention does not always happen.

### Strengths and Limitations

The current study has provided one of the first in-depth insights into students’ experiences of bullying at university. There may have been some issues with heterogeneity within the physical focus group as it was noted that one minoritized student may have been suppressed by a majority group member. However, this was countered using online groups, which were anonymous, and thus facilitated open expression. Structural power differences would have been unknown online and less likely to encroach upon responses, especially for minoritized voices on sensitive topics. Online groups were also found to provide an inclusive forum for those unable to physically attend a meeting. Additionally, it was unknown whether the reported opinions were personal experiences, experiences of friends, or hypothesised experiences of bullying within HE. This study aimed to gather a range of opinions and therefore did not record frequencies of personal bullying.

### Conclusions

This study explored students’ perceptions of bullying at HE level in the UK. Similarities and differences were seen between childhood bullying and bullying at university. The overarching theme of a power imbalance mirrors the school bullying literature, but there were subtle differences in how the power imbalances were perceived. In school, power imbalances often focus on physical factors such as size or age, whereas at university the power imbalances take the form of structural inequalities. In common with school bullying, the bullying could be goal-directed to gain hierarchical power, control, or status. However, due to the age of most students, bullying seems to adopt a more mature appearance and so harassment may be more disguised with hierarchies being more nuanced. There were some additional methods and tactics more commonly used at this level than in childhood, and these tactics aligned with those used within abusive romantic relationships. The study evidenced the advantage of approaching the research using a more inductive approach, thus broadening our understanding of bullying amongst EAs in a university context.

### Implications for HE Institutions

An important finding from this study relates to the propagation of systemic inequalities through bullying in HE, with minoritized groups being particularly vulnerable to victimisation. HE providers are legally obliged to protect these groups, and it is recommended that further consideration be given to these issues within bullying and harassment policies.

Additionally, we note that students consistently report difficulties in identifying bullying and knowing how to respond. We recommend that HE providers offer clear information to students, victims, and bystanders, to incorporate definitions, examples, and guidance on available support that more accurately reflects the experience of bullying within HE (see also Harrison, Fox, & Hulme, 2020).

The findings that relate to the role of the bystander support growing initiatives within schools and universities to encourage more individuals to become active, as opposed to passive bystanders. However, implementing change is not easy and requires widespread efforts to challenge norms that justify and minimise bullying, as well as helping witnesses overcome some of the barriers to intervening.

In conclusion, this study has made preliminary progress in providing a more nuanced understanding of bullying within HE that can inform future studies and efforts to tackle bullying within this context.
